# Risk factors for septic arthritis and multiple arthroscopic washouts: minimum 2-year follow-up at a major trauma centre

**DOI:** 10.1007/s10067-022-06151-w

**Published:** 2022-04-02

**Authors:** Victor Lu, Andrew Zhou, Hassan Abbas Hussain, Azeem Thahir, Matija Krkovic

**Affiliations:** 1 School of Clinical Medicine, University of Cambridge, Cambridge, CB2 0SP UK; 2 Christ’s College, St. Andrew’s Street, Cambridge, CB2 3BU UK; 3 Department of Trauma and Orthopaedics, Addenbrooke’s Hospital, Cambridge, CB2 0QQ UK

**Keywords:** Gout, Irrigation and debridement, Risk factors, Rheumatoid arthritis, Septic arthritis

## Abstract

**Background:**

Septic arthritis (SA) is a dangerous condition that requires emergency treatment. Managed by culture-specific antibiotics, irrigation, and debridement (I&D), some patients require repeat surgical treatment. The objectives were to determine the risk factors for SA and risk factors for repeat arthroscopic I&D in SA patients. We hypothesized that variables which directly or indirectly contributed to a larger infection burden would be associated with the development of SA and the need for repeat arthroscopic I&D.

**Methods:**

All patients ≥ 18 years old presenting to the emergency department, orthopaedic and rheumatology clinics at our major trauma centre between January 2018 and January 2020 with a hot, swollen joint were retrospectively evaluated. Patients with previous trauma and metalwork in the affected joint, periprosthetic joint infection, previous joint arthroplasty surgery, soft tissue infection, missing data, transferred to another centre, diagnosis not concerning the joint, and < 24-month follow-up were excluded. Two hundred eleven patients were included (SA: 28; pseudogout: 32; gout: 50; others: 101). Variables of interest in the 3-month period preceding the diagnosis of SA were compared between SA and non-SA patients using univariable analysis. A multivariable logistic regression model was formed using covariates with corresponding univariable tests of *p* < 0.200. Similar analyses were performed to compare SA patients with multiple washouts/procedures with those with one washout/procedure.

**Results:**

Multivariable analysis showed multiple risk factors for SA, namely rheumatoid arthritis (RA) (OR: 3.4; 95% CI: 1.2–10.0; *p* = 0.023); skin infection (OR: 3.3; 95% CI: 1.2–9.0; *p* = 0.017), liver disease (OR: 9.9; 95% CI: 2.2–43.9; *p* = 0.003), knee joint involvement (OR: 3.5; 95% CI: 1.3–9.4; *p* = 0.014), and use of immunosuppressive medication (OR: 3.5; 95% CI: 1.2–10.6; *p* = 0.027). Risk factors for multiple washouts included synovial WBC levels > 10.5 × 10^9^ cells/L (OR: 3.0; 95% CI: 2.3–38.8; *p* = 0.009) and RA (OR: 3.5; 95% CI: 1.9–66.3; *p* = 0.017).

**Conclusions:**

These findings suggest that prophylactic actions against septic arthritis should be targeted at patients with liver disease, RA, or skin infection. Repeat arthroscopic I&D of septic joints may be needed, especially in patients with synovial WBC levels > 10.5 × 10^9^ cells/L and RA.

**Table:** 

**Key Points** • *The risk factors for septic arthritis determined in this study are rheumatoid arthritis, skin infection, liver disease, knee joint involvement, and immunosuppressant usage.* • *Some septic arthritis patients need multiple rounds of arthroscopic irrigation and debridement. The risk factors for this are a synovial WBC count* > *10.5* × *10*^*9*^* cells/L and rheumatoid arthritis.*

**Supplementary Information:**

The online version contains supplementary material available at 10.1007/s10067-022-06151-w.

## Introduction

Septic arthritis (SA) is a medical emergency which has a mortality rate of 11% [1]. The treatment for SA involves irrigation and debridement (I&D) via arthroscopy or open arthrotomy to remove the microbial burden and any debris, combined with culture-specific antibiotics. A delay in diagnosis causes a delay in treatment, which leads to serious complications such as osteonecrosis and joint degradation [2]. There is controversy whether I&D should be performed arthroscopically or via open arthrotomy. Whilst Bovonratwet et al. detected no significant differences in minor adverse events and serious adverse events between the two treatment modalities [3], a therapeutic study over a 15-year time span concluded that arthroscopic treatment resulted in fewer I&D procedures and a higher cumulative success rate [4]. Further small-scale retrospective studies have concluded that arthroscopic treatment results in better functional outcomes [5, 6]. Local guidelines at the authors’ home institution suggest arthroscopic treatment as the first-line management for SA patients, in addition to culture-specific antibiotics.

The high morbidity and mortality rates of SA warrant an assessment of risk factors, which would facilitate clinicians in making a timely diagnosis. An important outcome is whether or not a patient needs repeated I&D treatment. Knowing the risk factors for an unplanned return to the operating theatre could allow clinicians to identify high-risk patients and optimize modifiable risk factors before treatment. We therefore studied the risk factors for septic arthritis in patients with the common presenting complaint of a hot, swollen joint, as well as the risk factors for repeat arthroscopic I&D. We hypothesized that covariates which directly or indirectly contributed to a larger infection burden would be associated with the development of SA and the need for repeat arthroscopic I&D.

## Methods

### Patient selection

Institutional board approval was received with the following project number: PRN10244. This retrospective study was performed according to the STROBE guidelines [7] (Supplementary Table 1). The inclusion criteria were all patients aged 18 years or older presenting to the emergency department, orthopaedic and rheumatology clinics at our major trauma centre between January 2018 and January 2020 with a hot, swollen joint. Patients were identified utilizing diagnostic coding provided by our institution’s electronic patient medical record system (ICD-10 codes). All patients’ diagnoses were confirmed by two rheumatologists or experienced orthopaedic surgeons, especially since joint swelling can be hard to define in the clinical setting in certain joints like the hip and shoulder.

The exclusion criteria included patients with previous trauma on the affected joint, previous joint arthroplasty surgery, treated at another centre before transferring to our institution, data missing from medical records in any of the variables required for analysis, soft tissue infection that required debridement (e.g. periarticular abscess or necrotizing fasciitis), metalwork in the affected joint, periprosthetic joint infection (PJI), a diagnosis that did not concern the joint per se (e.g. tenosynovitis, bursitis, cellulitis), or < 24 months follow-up. PJIs were excluded since they respond poorly to arthroscopic lavage, behave differently to native joints, and have important clinical differences with SA [8]. Plain radiographs were used to exclude other causes of joint swelling such as soft tissue trauma and joint fractures.

### Protocol

Patients were divided into four categories based on diagnostic findings: SA, gout, pseudogout, other conditions. SA was assessed with a mixture of clinical examination and laboratory findings. Patients were assessed for pyrexia, joint tenderness, erythema, effusion, and decreased range of movement. Laboratory findings looked for inflammatory marker elevation, with or without an elevated white blood cell (WBC) count. If SA was suspected, blood culture, arthrocentesis, and radiographical investigations were performed. Formal diagnosis of SA was made according to the criteria defined by Newman [9]. This involves a positive finding amongst the following four criteria: (1) positive synovial culture; (2) negative synovial culture but positive blood culture; (3) negative synovial culture but purulent joint discharge seen; (4) radiological evidence such as joint subluxation, periosteal reaction, osteolysis, and bony resorption [10].

All SA patients received arthroscopic I&D under general anaesthesia. After portals were inserted into the affected joint, infected joint material and synovial membrane biopsies were sent for culture. All affected joints were irrigated with high-volume arthroscopic lavage until the fluid was clear, with a powered shaver used to remove all visible fibrin deposits. Synovectomy was performed in seven patients with gross involvement of the synovial membrane (Gächter stage III/IV). A drain was left in situ after the portal sites were closed with a non-absorbable suture. Fluid from the drains was cultured on days 1, 3, and 5 after the procedure. Patients were started on two intravenous broad-spectrum antibiotics, usually vancomycin (15–20 mg/kg every 12 h) and tazocin (4.5 g every 8 h). After multidisciplinary team discussions with the infectious disease consultants and orthopaedic surgeons, antibiotics were made culture-specific based on microorganisms’ sensitivities. All patients were offered range-of-motion exercises to prevent joint stiffness. Repeat arthroscopic I&D was a discretionary treatment decision based on any of the following signs: worsening physical symptoms (e.g. increasing pain, decreased range of motion), post-washout culture results, persistent purulent drainage from the joint, continuously elevated inflammatory markers, and was made by a joint decision between one orthopaedic surgeon and one infectious disease specialist to reduce inter-observer variability and increase internal validity. The average interval between arthroscopic I&Ds was 120 (range 40–242) h. Any procedure that was performed more than 2 weeks after the prior procedure was deemed independent to the prior event, and the patient was assigned to the ‘one washout’ group.

Examination and laboratory findings for the SA cohort and pseudogout cohort are presented in Tables 1 and 2. Immunosuppressant medication was defined as prednisolone, methotrexate, tacrolimus, or azathioprine.

**Table 1:** Examination findings of septic arthritis patients

CRP (mg/L)	WBC (× 10^9^ cells/L)	Organism	Age	Warm	Tender	Swollen	Effusion	Redness	Fever	Movement	Multiple washouts
152	12.4	*Clostridium septicum*	75	Y	Y	Y	N	N	N	Reduced	No
155	23.7	*E. coli*	60	N	Y	N	Y	Y	N	Reduced	Yes
245	15.3	MSSA	78	N	Y	Y	N	Y	N	Reduced	No
95	9.6	MSSA	86	Y	N	Y	N	N	Y	Reduced	No
158	10.7	MSSA	26	Y	Y	Y	Y	N	Y	Reduced	No
99	10.4	*Clostridium septicum*	50	Y	N	Y	Y	Y	N	Reduced	No
19.4	15.4	Gramme − ve bacillus	60	N	N	N	N	N	Y	Reduced	No
249	6.7	MSSA	73	N	N	Y	N	N	N	Reduced	Yes
481	21	MSSA	82	N	Y	Y	N	N	Y	Reduced	Yes
252	24	MSSA	68	Y	N	Y	Y	N	N	Reduced	No
139	11.3	MSSA	47	N	Y	N	N	N	Y	Reduced	Yes
334	10.4	MSSA	38	N	N	N	Y	N	Y	Reduced	No
376	10.7	*Strep pneumoniae*	54	N	Y	N	N	N	N	Reduced	No
304	9.7	Gramme − ve bacillus	63	N	Y	N	Y	N	N	Reduced	No
31	12.5	Gramme − ve bacillus	81	Y	Y	Y	N	N	Y	Reduced	Yes
347	8.8	MSSA	35	N	Y	N	Y	N	N	Reduced	No
328	28.1	MSSA	86	N	N	N	N	N	N	Reduced	Yes
453	15.2	MSSA	51	N	N	Y	N	N	Y	Reduced	Yes
245	23.6	*E. coli*	27	N	N	N	Y	Y	N	Reduced	Yes
154	12.3	MSSA	78	Y	Y	Y	N	N	Y	Reduced	Yes
357	10.6	MSSA	65	N	N	Y	Y	N	N	Reduced	No
343	9.7	*Strep pneumoniae*	85	Y	Y	Y	N	N	Y	Reduced	No
323	21.1	MSSA	87	N	Y	N	Y	Y	N	Reduced	Yes
27	24.3	*Strep pneumoniae*	54	Y	Y	Y	Y	N	Y	Reduced	No
342	16.7	MSSA	82	N	Y	N	N	N	N	Reduced	Yes
323	10.4	MSSA	80	N	N	N	N	N	Y	Reduced	No
289	10.5	MSSA	34	N	N	Y	N	N	N	Reduced	No
389	10.6	*Strep pneumoniae*	42	N	Y	N	Y	N	N	Reduced	No
250.3 ± 128	14.5 ± 5.9	MSSA: 60.7%	62.4 ± 19.5	Yes: 32.1%	Yes: 57.1%	Yes: 57.1%	Yes: 42.9%	Yes: 17.9%	Yes: 42.9%	Reduced: 100%	Yes: 39.3%

*WBC*, white blood cell; *CRP*, C-reactive protein; *MSSA*, methicillin-susceptible *Staphylococcus aureus*

**Table 2:** Examination findings of pseudogout patients

CRP (mg/L)	WBC (× 10^9^ cells/L)	Age	Warm	Tender	Swollen	Effusion	Redness	Fever	Movement
267	15.8	86	N	N	Y	N	N	Y	Reduced
69	4.9	78	Y	N	Y	N	N	N	Reduced
91.5	22	74	N	N	N	N	N	N	Normal
187	12.6	89	N	N	Y	Y	N	Y	Reduced
226	15	90	Y	N	N	Y	N	Y	Reduced
66	17.7	68	N	Y	Y	Y	N	N	Reduced
117	10.8	85	N	Y	Y	Y	N	N	Reduced
42.3	10.8	93	Y	Y	N	N	N	N	Reduced
249	15.9	93	N	N	Y	N	N	N	Reduced
226	15.6	91	Y	Y	Y	Y	N	N	Reduced
99	10.4	70	Y	Y	Y	Y	N	Y	Reduced
98.4	7.37	57	Y	N	Y	Y	N	N	Reduced
39.2	10.3	86	Y	N	Y	N	Y	N	Reduced
94	12.6	75	Y	N	Y	Y	N	N	Reduced
323	6.4	93	N	N	Y	N	N	N	Reduced
28	14.4	77	N	N	N	N	N	Y	Normal
95	11.6	72	Y	Y	Y	N	Y	N	Reduced
333.9	16.1	73	Y	N	Y	Y	N	Y	Reduced
63	10	90	N	N	N	Y	N	N	Normal
37	3.2	93	N	N	N	N	N	Y	Normal
66	10.4	78	Y	N	Y	Y	N	N	Reduced
22	6	58	N	Y	N	Y	Y	N	Reduced
38	12.5	78	Y	N	Y	N	N	N	Reduced
181	11.4	85	Y	N	Y	N	N	N	Reduced
292	14	84	Y	Y	N	Y	N	Y	Reduced
35	7.5	91	Y	N	N	Y	N	N	Reduced
42	9	77	Y	N	N	Y	N	N	Reduced
34	11.1	61	Y	Y	Y	Y	N	N	Reduced
160	7.9	82	Y	Y	Y	N	N	Y	Reduced
92	3.1	64	N	N	Y	Y	N	N	Reduced
174	5.8	78	Y	Y	N	Y	N	N	Reduced
4	6.1	72	Y	N	Y	N	N	N	Reduced
121.6 ± 95.7	10.9 ± 4.4	79.4 ± 10.6	Yes: 62.5%	Yes: 34.3%	Yes: 65.6%	Yes: 56.2%	Yes: 9.4%	Yes: 28.1%	Reduced: 87.5%

*WBC*, white blood cell; *CRP*, C-reactive protein; *MSSA*, methicillin-susceptible *Staphylococcus aureus*

### Data analysis

To identify risk factors for SA, patients with SA were compared to patients who presented with a hot swollen joint, but did not have SA, according to the aforementioned inclusion and exclusion criteria. Age was dichotomized (≥ 80 vs < 80) to streamline interpretation, with the value 80 chosen based on previous literature [11, 12]. SA patients with multiple arthroscopic I&Ds were also compared to SA patients with one arthroscopic I&D, to determine risk factors for multiple arthroscopic I&Ds.

Statistical analysis was performed using IBM SPSS Statistics version 28.0. Univariable analysis was performed to identify prognostic factors for SA. Covariates with a *p*-value of *p* < 0.200 in the univariable analysis were included in a multivariable logistic regression model, which then underwent a backward elimination process to identify the best predictive model for SA. An intercorrelation matrix was made using RStudio version 4.0.5 to assess for multi-collinearity between covariates. The same methodology was used when analyzing risk factors for multiple arthroscopic I&Ds in patients with SA.

## Results

**Fig. 1:**
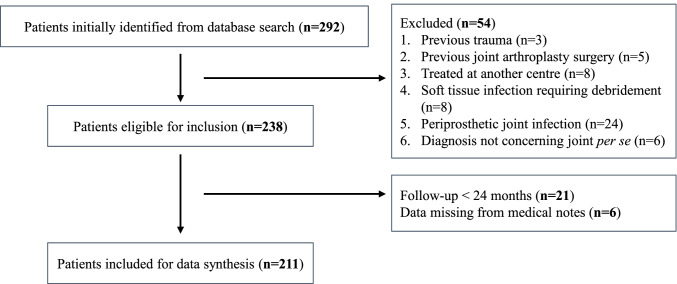
Flow diagram showing number of individuals at each stage of the study

A total of 211 patients were identified for analysis. Twenty-eight patients had the primary diagnosis of SA, 32 had pseudogout, 50 had gout, and 101 had other conditions (Table 3). The distribution of joints affected is shown in Table 4. The mean follow-up time was 2.86 years (range 2.01–4.17 years). Out of the 292 patients initially identified, 21 (7.19%) did not meet the minimum 2-year follow-up period (Fig. 1).

**Table 3:** Diagnosis of patients presenting with a hot, swollen joint

Total	211
Septic arthritis	28
Pseudogout	32
Gout	50
Others	101
Lupus arthritis	9
Osteoarthritis	19
Psoriatic arthritis	11
Palindromic rheumatism	5
Chronic joint effusion	15
Haemathrosis	10
Rheumatoid arthritis	18
Undifferentiated arthritis	12
Ankylosing spondylitis	2

**Table 4:** Distribution of joints affected

Joint	Number of Joints
Septic arthritis	
Knee	18
Shoulder	8
Hip	2
Pseudogout	
Knee	10
Shoulder	2
Elbow	3
Wrist	9
Ankle	8
Gout	
Knee	15
Elbow	4
Wrist	7
Ankle	9
Metatarsophalangeal	15
Other pathologies	
Knee	40
Shoulder	18
Hip	14
Sternoclavicular	2
Carpometacarpal	3
Ankle	12
Wrist	12

**Fig. 2:**
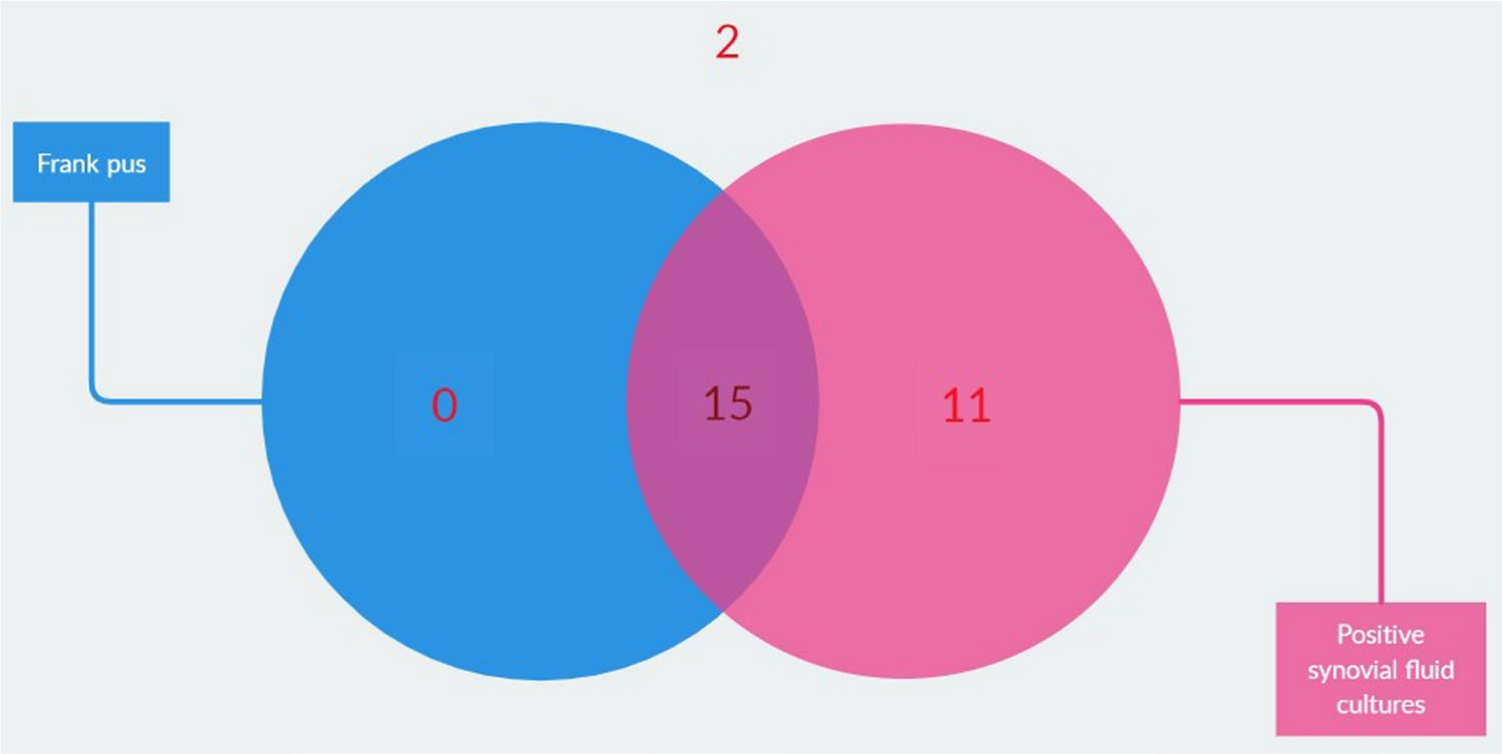
Distribution of septic arthritis patients with frank pus or positive synovial fluid cultures. The two patients without positive synovial fluid cultures or frank pus had radiographical indications and positive cultures from surgical samples

The observation of frank pus removed any suspicion about the underlying pathology; however, this was only present in fifteen SA patients (Fig. 2). Blood cultures were performed in fifteen patients, of which ten returned positive. Six of these patients also had a positive synovial fluid culture. Six patients received antibiotics prior to joint aspiration, two of which returned negative synovial fluid cultures. Nevertheless, they had radiographical indications such as subchondral erosions, perisynovial oedema, and positive cultures from surgical samples taken during arthroscopic treatment, confirming the diagnosis of SA.

### SA risk factors

Univariable regression analysis identified several prognostic factors for SA (Table 5). Age ≥ 80 (*p* = 0.090), diabetes mellitus (*p* = 0.076), smoking (*p* = 0.060), liver disease (*p* = 0.017), rheumatoid arthritis (*p* = 0.033), use of immunosuppressants (*p* = 0.171), involvement of the knee joint (*p* = 0.112), and skin infection (*p* = 0.152) were associated with SA and considered for the multivariable logistic regression model.

**Table 5:** Univariable and multivariable logistic regression analyses

*N* = 211	Septic arthritis					
	Univariable			Multivariable		
	Septic arthritis (%; *n* = 28)	Non-septic arthritis (%; *n* = 183)	*p*-value	Odds ratio	95% CI	*p*-value
Age ≥ 80	8 (28.6)	22 (12.0)	*0.049*			
BMI	26.01	26.33	0.962			
Male gender	14 (50.0)	83 (45.4)	0.408			
Diabetes mellitus	10 (35.7)	29 (15.8)	*0.046*			
Smoking	10 (35.7)	35 (19.1)	*0.025*			
Alcohol	12 (42.9)	70 (38.3)	0.758			
Liver disease	5 (17.9)	6 (3.3)	*0.005*	9.859	2.217–43.854	*0.003*
Cardiac disease	1 (3.6)	11 (6.0)	0.322			
Renal and urological disease	1 (3.6)	8 (4.4)	0.919			
Malignancy	0 (0)	3 (1.6)	0.999			
Joint pathology						
RA	14 (50.0)	28 (15.3)	*0.023*	3.424	1.186–9.889	*0.023*
AS	0 (0)	2 (1.1)	0.999			
OA	2 (7.1)	19 (10.4)	0.570			
Immunosuppressants	12 (42.9)	20 (10.9)	*0.172*	3.498	1.154–10.602	*0.027*
Knee involvement	18 (64.3)	65 (35.5)	*0.006*	3.472	1.281–9.410	*0.014*
Infection						
Skin	12 (42.9)	26 (14.2)	*0.107*	3.344	1.241–9.011	*0.017*
Respiratory tract	5 (17.9)	28 (15.3)	0.261			
UTI	2 (7.1)	10 (5.5)	0.251			
*N* = 28	Repeat I&D in septic arthritis patients					
	Univariable			Multivariable		
	Repeat washout (%; *n* = 11)	One washout (%; *n* = 17)	*p*-value	Odds ratio	95% CI	*p*-value
Age ≥ 80	3 (27.3)	5 (29.4)	0.903			
Immunosuppression	8 (72.7)	4 (23.5)	*0.010*			
Male gender	6 (54.5)	8 (47.1)	0.699			
Diabetes mellitus	3 (27.3)	7 (41.2)	0.453			
Smoking	4 (36.4)	4 (23.5)	0.954			
RA	9 (81.8)	5 (29.4)	*0.007*	3.52	1.867–66.305	*0.017*
Alcohol	3 (27.3)	9 (52.9)	0.180			
Synovial WBC above 10.5 × 10^9^ cells/L	10 (90.9)	8 (47.1)	*0.018*	3.00	2.328–38.761	*0.009*
BMI	24.5 ± 4.8	22.8 ± 3.9	0.353			
Knee involvement	10 (90.9)	8 (47.1)	*0.018*			
Synovectomy	3 (27.3)	4 (23.5)	0.823			

*BMI*, body mass index; *RA*, rheumatoid arthritis; *OA*, osteoarthritis; *AS*, ankylosing spondylitis; *UTI*, urinary tract infection; *WBC*, white blood cell; *I&D*, irrigation and debridement; *p*-values < 0.200 in univariable analysis that were considered for the multivariable model are italicised; *p*-values <0.05 in the multivariable model are italicised

A multi-collinearity test detected intercorrelation between the variables smoking, age ≥ 80, and diabetes mellitus (*r* ≥ 0.8). The multivariable regression model with those variables removed produced the highest Nagelkerke R square value and was hence used. This showed that the risk factors for SA were rheumatoid arthritis (RA) (OR: 3.4; 95% CI: 1.2–10.0; *p* = 0.023); skin infection (OR: 3.3; 95% CI: 1.2–9.0; *p* = 0.017), liver disease (OR: 9.9; 95% CI: 2.2–43.9; *p* = 0.003), knee joint involvement (OR: 3.5; 95% CI: 1.3–9.4; *p* = 0.014), and use of immunosuppressive medication (OR: 3.5; 95% CI: 1.2–10.6; *p* = 0.027) (Table 5).

### Risk factors for multiple arthroscopic I&Ds in SA patients

Univariable regression analysis identified prognostic factors for repeated arthroscopic I&Ds in SA patients. All covariates had a higher incidence in those that required multiple arthroscopic I&Ds, except for proportion of smokers, percentage of males and diabetics, and alcohol consumption.

RA and synovial WBC > 10.5 × 10^9^ cells/L were included in the multivariable regression model and multi-collinearity analysis detected no intercorrelation (Table 5). Both were risk factors for multiple washouts, with odds ratios of 3.00 (95% CI: 2.328–38.761; *p* = 0.009) and 3.52 (95% CI: 1.867–66.305; *p* = 0.017), respectively.

## Discussion

A hot swollen joint may progress insidiously, or rapidly deteriorate with disastrous consequences [10]. When discovered in time, SA is no cause for alarm; however, a large proportion of diagnoses in the ED are made late [11]. Furthermore, SA can sometimes be subtle, and have been mistaken for other conditions such as a periprosthetic joint infection (PJI) [8]. Kandoorp et al. presented the risk factors of septic arthritis in a cohort of patients with pre-existing joint disease [13]. Yet to our knowledge, the risk factors for septic arthritis in patients with the common presenting complaint of a hot, swollen joint have not been analyzed. Understanding the risk factors for SA in this cohort of patients is beneficial for clinical practice as it allows the clinician to take a detailed history with emphasis on specific risk factors discussed in this study. This allows for prompt diagnosis and treatment. Using our large sample size, this study additional seeks to determine the risk factors associated with multiple washouts, which allows the development of a prognostic algorithm in clinical practice. Our results showed that RA, skin infection, liver disease, and knee joint involvement, and use of immunosuppressive medication were risk factors for SA. A synovial WBC count exceeding 10.5 * 10^9^ cells/L and RA were risk factors for multiple arthroscopic I&D treatments.

### SA risk factors

With older age, more comorbidities arise, some of which can increase the risk of joint infection, such as arthritis, arthropathies, chronic diseases requiring immunosuppression, or a general decrease in immunocompetence [14]. We found that older age is associated with SA independent of other risk factors such as RA or skin infection. Nevertheless, other covariates such as OA also has a relationship with age, with two-thirds over the age of sixty having OA [15]. Despite gender not being a risk factor (*p* = 0.417), we found that more females over the age of 80 (87.5%) have SA than males (12.5%), which could be due to the longer life span in females [16]. Particularly in the elderly, infection is sometimes not the primary symptom, with 19% of patients over 60 [17] and 23% of patients over 80 [18] remaining afebrile in the literature, and 50% of patients above 80 in our cohort remaining afebrile. This is important to note since SA in those over 85 was found to have an adjusted hazard ratio for increased mortality of 1.79 (95% CI: 1.59–2.02) [12]. The only patient over 80 with a normal synovial WBC count in our cohort had diabetes but a severely reduced range of motion in the affected joint.

A common theme amongst the variables identified as risk factors for SA is the heightened risk for infection. It was not surprising that diabetes and the use of immunosuppressive medications were associated with SA. Diabetes leads to immune system dysfunction, by reducing phagocytotic ability of macrophages and impairing activation of the adaptive immune system [19]. SA located in rarely reported joints and caused by microorganisms that rarely infect humans occurs in those with diabetes. In a case series of all reported cases of acromioclavicular joint SA in the literature, 20% were diabetics and 25% had a suppressed immune system [20]. A diabetic was reported to have SA due to *Sphingomonas paucimobilis*, which led to septic pulmonary emboli [21]. A diabetic farmer presented with left ankle SA due to *Actinomyces pyogenes* infection, which is usually found in cattle [22]. Nevertheless, some type 2 diabetes medications such as the dipeptidyl peptidase-4 inhibitors have reported to lead to a hot swollen joint with severe pain that mimics SA [23]. Given that diabetes is also an independent predictor of mortality in SA patients [12], it is important to optimize modifiable risk factors during and after treatment.

It is well-known in the literature that large joints are more susceptible to SA [3, 24]. We found that the knee joint was an independent risk factor for SA. The lack of a basement membrane in the vessels of the synovial intima [25], the synovial lymphatic system [26], and the dense vascularity all encourage haematogenous inoculation of the knee joint. Pre-existing joint damage has been reported as a risk factor for SA, due to neovascularization and increased adhesion factors promoting bacteraemia [13]. Joint structure abnormality can allow pathogens to escape phagocytosis, and infected joints undergo more rapid histological changes [27]. However, only RA, rather than ankylosing spondylitis (AS) or OA, was found to be a risk factor. This could be because the RA patient has concurrently the greatest number of other risk factors for SA, such as old age [28], diabetes mellitus [29], skin infection [5], and liver disease [30]. RA significantly increases the risk of SA, with an incidence rate of 70 cases per 100,000 person-years compared to 2–5 cases per 100,000 person-years [24]. Given that immunosuppressants are an independent risk factor for SA, it could be argued that RA is a risk factor due to the patients taking immunosuppressants. However, 3/13 (23.1%) of RA patients in our SA cohort did not take steroids or DMARDs. Anti-tumour necrosis factor (TNF) therapy has been reported to double the risk of SA [31]; however, no RA patients in our cohort were on anti-TNF therapy. Like diabetics, RA patients have a lower infection resistance, with an increased mortality from infectious diseases [32]. Furthermore, pathogens from skin lesions and infections, which we found to be an independent risk factor for SA, could travel to neighbouring lymph nodes via an inflamed synovium. Where a source could be found, the skin accounted for 76% of cases of SA in the literature [33]. RA patients with SA are often diagnosed late due to confusion with a relapse of underlying joint pathology [24], making joint culture and microbiological analysis crucial for timely diagnosis in this patient cohort.

Liver disease in musculoskeletal patients is not well-described in the literature. Several case reports and case series describe septic arthritis in patients with liver cirrhosis [34–37]. Liver cirrhosis can lead to neutrophil phagocytic dysfunction and impairment of the innate immune system, which leads to increased risk of infection [34]. In our cohort, three out of five patients had liver cirrhosis; the other two had fatty liver disease, all of which had moderate alcohol consumption. Nevertheless, our study did not find alcohol to be an independent risk factor for SA. Only one study has reported a connection between chronic alcohol abuse and SA [38]. Alcohol is associated with increased rates of skin infection [39], with 7/12 (58.3%) alcoholics in our cohort having skin infections, as well-increased risk of MSSA infection [39], with 5/12 (41.7%) alcoholics in our cohort having an MSSA infection. Portal hypertension can put patients at risk of spontaneous bacteraemia and spontaneous bacterial arthritis [35]. Despite the *p*-value of 0.003, the number of alcoholics and patients with liver cirrhosis in our cohort is small, and more research is needed to confirm the relationship between liver disease, alcoholism, and SA. Nevertheless, given that liver disease is also an independent predictor of mortality in SA patients [12], we urge clinicians to consider the possibility of SA in cirrhotic patients with pyrexia in whom no other cause can be found, particularly in less accessible joints.

### Risk factors for multiple arthroscopic I&Ds in SA patients

The incidence of repeat washouts in our cohort (39.3%) is similar to the existing literature citing 41% [40]. Other studies have investigated a similar topic, with variables such as diabetes [2], positive drainage-fluid culture [41], and a concurrent inflammatory arthropathy (IA) [2]. Although our cohort was too small to investigate other IAs, RA was a risk factor for arthroscopic washout failure, with the underlying pathophysiology similar to how RA is a risk factor for SA. Although eliminated after stepwise regression in our multivariable model, the knee joint was also found to be associated with treatment failure. This agrees with Hunter et al. who found that a large joint (knee, shoulder, hip) was an independent predictor of treatment failure (OR: 7.3; 95% CI: 2.4–22.6; *p* < 0.001). A proposed culprit for increased risk of persistent infection after arthroscopic management is the synovium. Hyper-vascularization post-infection and the accumulation of bacteria-laden phagocytes in the synovial membrane forms a niche for persistent infection, with thorough synovectomy needed to reduce bacterial burden. However, no significant association with treatment failure was found (*p* = 0.823). Furthermore, a thorough synovectomy prolongs the intraoperative time and may require accessory portals [42].

The identification of an elevated synovial WBC count and RA as risk factors allows the risk stratification of patients and the opportunity for additional interventions to lower the chance for returning to the operating theatre. An elevated synovial WBC count could suggest persistent infection, either at the joint itself due to inadequate washout, or somewhere else in the body, which continue to seed bacteria haematogenously into the synovial fluid. Future research could determine if the pathogen causing a concurrent infection and the septic joint is the same or not.

### Limitations

Our study was retrospective in design, and there was no formal clinical definition for whether or not a repeat washout is needed, with this decision being made at the clinician’s discretion. Yet internal validity was increased and bias minimalized by reducing the number of clinicians making the decision for a repeat washout, and decisions were based on robust clinical and pathological signs such as persistently elevated inflammatory markers, worsening physical symptoms, and positive drainage-fluid culture. Nevertheless, external validity may still be an issue, given that other clinicians in other centres will be making the decision at their own discretion.

The 21 patients lost to follow-up may have introduced a nonresponse bias, with nonparticipants having different characteristics than participants. Fortunately, epidemiological studies have found little effect of nonresponse bias on the outcomes of a study [43]. The number of patients coming from a rheumatology or orthopaedic clinic was similar, decreasing the chance that there was an over-representation of patients with inflammatory arthritis. There was also no standardized protocol for antibiotic selection, with varying culture sensitivities and patient allergies complicating the generation of antibiotic regimens. Although unable to control for antibiotic therapy and medical compliance in patients, we believe that this is a realistic representation of clinical practice in this patient group. Furthermore, some patients with only one arthroscopic I&D could have had another one done elsewhere which was not documented, but this possibility is slim. Finally, whilst our data delivers prognostic information on the risk for SA and repeat arthroscopic I&Ds, it does not determine the patients’ prognosis. More research is needed to corroborate our results and determine if they are also associated with the outcome of patients’ hospital stay, and their subsequent functional status.

## Conclusion

With a high mortality and morbidity rate, early diagnosis of septic arthritis is crucial, which can also impact the therapeutic outcome. This requires understanding what a typical patient presents with: a warm, erythematous, tender knee joint with either a history of rheumatoid arthritis, skin infection, or liver disease. A high WBC count and concurrent RA increase the risk of arthroscopic treatment failure. We believe that the models in this study are of prognostic value to clinicians who are presented with the common presenting compliant of a hot swollen joint. The identification of specific risk factors could lead to improved prevention and risk stratification tools.

## Supplementary Information

Below is the link to the STROBE Guidelines.

STROBE Guidelines (DOCX 35 KB)
